# Comparison of the Surface Properties of Hydrothermally Synthesised Fe_3_O_4_@C Nanocomposites at Variable Reaction Times

**DOI:** 10.3390/nano11102742

**Published:** 2021-10-16

**Authors:** Sadiq Sani, Rohana Adnan, Wen-Da Oh, Anwar Iqbal

**Affiliations:** 1 School of Chemical Sciences, Universiti Sains Malaysia (USM), Penang 11800, Malaysia; sadiqsani123@gmail.com (S.S.); ohwenda@usm.my (W.-D.O.); anwariqbal@usm.my (A.I.); 2 Department of Applied Chemistry, Federal University Dutsin-Ma, Dutsinma P.M.B. 5001, Nigeria

**Keywords:** ANOVA model, heating temperature, hydrothermal synthesis, magnetic nanocomposite, reaction time, surface properties

## Abstract

The influence of variable reaction time (t_r_) on surface/textural properties (surface area, total pore volume, and pore diameter) of carbon-encapsulated magnetite (Fe_3_O_4_@C) nanocomposites fabricated by a hydrothermal process at 190 °C for 3, 4, and 5 h was studied. The properties were calculated using the Brunauer–Emmett–Teller (BET) isotherms data. The nanocomposites were characterised using Fourier transform infrared spectroscopy, X-ray diffraction analysis, thermogravimetry, and scanning and transmission electron microscopies. Analysis of variance shows t_r_ has the largest effect on pore volume (F value = 1117.6, *p* value < 0.0001), followed by the surface area (F value = 54.8, *p* value < 0.0001) and pore diameter (F value = 10.4, *p* value < 0.001) with R^2^-adjusted values of 99.5%, 88.5% and 63.1%, respectively. Tukey and Fisher tests confirmed t_r_ rise to have caused increased variations in mean particle sizes (11–91 nm), crystallite sizes (5–21 nm), pore diameters (9–16 nm), pore volume (0.017–0.089 cm^3^ g^−1^) and surface area (7.6–22.4 m^2^ g^−1^) of the nanocomposites with individual and simultaneous confidence limits of 97.9 and 84.4 (p-adj < 0.05). The nanocomposites’ retained Fe–O vibrations at octahedral (436 cm^−1^) and tetrahedral (570 cm^−1^) cubic ferrite sites, modest thermal stability (37–60 % weight loss), and large volume-specific surface area with potential for catalytic application in advanced oxidation processes.

## 1. Introduction

In recent years, concerted efforts have been made to develop magnetite (Fe_3_O_4_) nanocomposite materials with improved surface properties suitable for specific applications in diverse fields. As nontoxic, biocompatible, and superparamagnetic materials of relatively high chemical stability, Fe_3_O_4_ nanocomposites find applications in biotherapy and biomedicine [1,2], drug targeting and delivery [3,4], catalysis [5,6,7], energy storage and release [8,9], environmental remediation [10], ultrafiltration membrane separation [11] and microwave absorption [12,13].

Fabrication of carbon-encapsulated magnetite nanocomposite materials (Fe_3_O_4_@C) by hydrothermal treatment is a popular method of rendering prevention to the parent Fe_3_O_4_ NPs from agglomeration and deterioration in chemical stability, thereby maintaining their effective surface properties, making them compatible for applications in both inorganic and organic processes. It involves synthesising Fe_3_O_4_@C nanostructures by heating at reaction temperatures and pressures above the ambient conditions of boiling water for a given reaction time, with the mixture of precursors in aqueous media as reported in the literature [14,15]. The method depends on precursor concentrations, the nature of the aqueous solvent, the stabilising agent, the type of precursor, the heating temperature, and the reaction time, which considerably influence the final products. It gives relatively low product yields compared to the co-precipitation that produces nanocomposites with a weak crystal structure [16,17]. However, unlike the “low temperature” co-precipitation technique, the temperature–pressure synergistic effect in the hydrothermal approach offers a one-step route for producing magnetic nanocomposites of high crystallinity and removing the need for post-annealing [18,19].

However, several works have reported hydrothermal treatments using one-off levels of the process parameters (reaction temperature, reaction time, etc.) without setting them at variable levels to optimise their influences and compare their efficiencies in the fabrication of Fe_3_O_4_@C nanocomposites with defined surface characteristics. For example, several heating temperature–reaction time pairs reported for hydrothermal synthesis of Fe_3_O_4_@C include 180 °C for 14 h [20], 170 °C for 4 h [21], 600 °C for 4 h [22], 210 °C for 48 h [23],160 °C for 10 h [24] and 180 °C for 24 h [25]. One-factor-at-a-time (OFAT) design optimisation of a hydrothermal process with few parameters furnishes optimal responses that are more reliable, minimises costs and maximises the utility of resources required to run the experiments. The lower the number of input parameters, the fewer are the runs and the resources for the experiments [26].

Therefore, fewer efforts were channelled into the use of OFAT design to optimise the effects of a limited number of independent factors (e.g., reaction time, temperature, amounts of Fe_3_O_4_ NPs precursor, amount of glucose precursor, etc.) on the surface characteristics of the carbon-encapsulated magnetite nanocomposites obtained via conventional hydrothermal synthesis. For instance, Subramanian et al. [27] accomplished the hydrothermal synthesis of MnO_2_ by starting with well-mixed aqueous solutions of MnSO_4_·H_2_O and KMnO_4_ by loading into an oven preheated at 140 °C to evaluate the influence of variable reaction time (1–18 h) on the end material.

Thus, the main objectives of this work are to conduct the hydrothermal synthesis of Fe_3_O_4_@C composite samples and to statistically compare the dependence of their selected surface properties (surface area, total pore volumes and pore diameters) on variable reaction time at a fixed temperature using one-way analyses of variance (ANOVA). Based on the data gathered from the literature, three different reaction times were selected (3, 4, and 5 h). Tukey simultaneous tests and Fisher individual tests were also conducted to determine the differences in the means of the properties for comparison. The reaction time dependent mesoporosity, monodispersity, shape controllability, and stability of the as-synthesised Fe_3_O_4_@C nanocomposite samples were further ascertained by performing morphological and structural elucidations using suitable instrumental analyses.

## 2. Materials and Methods

### 2.1. Materials

Nitric acid (HNO_3_), ethanol (C_2_H_5_OH, 95 %), anhydrous (D+)-glucose (C_6_H_12_O_6_) and ethylenediamine ((H_2_NCH_2_)_2_) were supplied by HmbG Chemicals, Kuala Lumpur, Malaysia. Ethylene glycol ((HOCH_2_)_2_), sodium acetate (CH_3_COONa) and ferric chloride hexahydrate (FeCl_3_·6H_2_O) were purchased from Bendosen Laboratory Chemicals, Kuala Lumpur, Malaysia. The as-received analytical grade pure chemical reagents and deionised water were used in the entire experimental procedures. 

### 2.2. Synthesis of Fe_3_O_4_@C Nanocomposite

Following the procedure mentioned elsewhere in the literature, mesoporous Fe_3_O_4_@C nanoparticles were prepared using a two-step process involving solvothermal and hydrothermal processes [28,29]. A solvothermal approach was first utilised to fabricate magnetite NPs. Weighed amounts of FeCl_3_·6H_2_O (1.0 g) and anhydrous CH_3_COONa (4.0 g) were dissolved in a mixture of ethylene glycol (27 mL) and ethylenediamine (3 mL) under vigorous magnetic stirring at 1000 rpm for at least 30 min to form a homogenised clear yellow solution. The resulting solution was heated at 220 °C for 2 h in a 50mL tightly sealed, Teflon-lined stainless-steel autoclave before cooling to room temperature under a cold tap water jet. The as-synthesised black magnetite nanoparticles were separated from the mixture using a magnet, washed copiously six times in a row with deionised water and ethanol, oven dried at 70 °C for 12 h under vacuum, and stored in an air-tight sample bottle placed in a desiccator for further processing. 

The synthesis of the Fe_3_O_4_@C nanocomposites was carried out by first immersing 0.23 g (0.001 mol) of Fe_3_O_4_ NPs in 0.1 M HNO_3_ solution for 5 min. Then, the nanoparticles were separated using a magnet and washed three times with deionised water. Then, the Fe_3_O_4_ NPs and 3.61 g (0.02 mol) of glucose were dissolved in 40 mL of deionised water under vigorous stirring for at least 10 min to homogenise the mixture. The mixture was oven-heated at 190 °C for the reaction time (t_r_) of 3 h in a 50 mL Teflon-sealed autoclave in an oven. The as-obtained magnetic composite was then allowed to cool naturally, separated with a magnet, washed three times with deionised water and ethanol and oven-dried at 60 °C for 12 h under vacuum. The same procedure was repeated two more times under the same hydrothermal conditions for t_r_ values of 4 and 5 h. The three Fe_3_O_4_@C nanocomposites obtained were labelled as Fe_3_O_4_@C-T_190_t_3_, Fe_3_O_4_@C-T_190_t_4_ and Fe_3_O_4_@C-T_190_t_5_.

### 2.3. Instrumental Analyses

The characteristic chemical bonds in the Fe_3_O_4_@C-T_190_t_3_, Fe_3_O_4_@C-T_190_t_4_ and Fe_3_O_4_@C-T_190_t_5_ nanocomposites were elucidated in the range of 400–4000 cm^−1^ using a Fourier transform infrared (FTIR) spectrophotometer (Perkin Elmer System 2000 FTIR). The powdered samples of translucent pellets were prepared by mixing the sample with dry KBr followed by hydraulic compression and then inserted into the sample holder of the spectrophotometer for analyses. 

The XRD tests of the as-synthesised nanocomposites samples were carried out on a fully automated X-Ray Diffractometer (Bruker’s D8 Advance, Bruker Corporation, Billerica, MA, USA) located at the Makmal Pencirian Bahan Bumi, Centre for Global Archaeological Research, Universiti Sains Malaysia. Powdered samples were first prepared in the shape of a circular disc (25 mm diameter) by hydraulic compression, followed by insertion into the sample holder for examination. Mounted flat on a glass slide, the circular discs were gently squeezed parallel to the holder’s surface. The XRD tests were conducted at a scanning velocity of 0.04 °C sec^−1^ for 25 min using the Cu-K_α_ radiation source (λ = 1.54060 Å), 40 kV voltage, 40mA current and 2θ scanning range of 10–70° at room temperature (25 °C). 

Datasets on thermogravimetric analysis (TGA) and derivative thermogravimetry (DTG) were collected on a thermogravimetric analyser (Perkin Elmer STA 6000 model, PerkinElmer Inc., Waltham, MA, USA), domiciled at the School of Chemical Sciences, Universiti Sains Malaysia. The analyses were conducted by heating in air at 80 mL min^−1^. The heating was carried out from 30 to 900 °C at a rate of 10 °C min^−1^, followed by holding the temperature for 5.0 min at 900 °C. 

A scanning electron microscope (Quanta FEG 650 SEM, FEI Company, Hillsboro, OR, USA) fitted with an energy-dispersive X-ray spectroscopy method (Oxford X-Max 50 mm^2^ EDX, Oxford Instruments, Oxford, UK) was used to investigate the morphology and elemental analysis of the nanocomposites. The surface morphologies of the nanocomposites were further observed by employing an energy-filtered transmission electron microscope (Zeiss Libra 120, Carl Zeiss NTS GmbH, Oberkochen, Germany). The resulting energy-filtered transmission electron microscopy (EFTEM) micrographs were used to study the particle size distribution of the nanocomposites. The samples were sonicated in ethanol for 5 min to prepare ethanolic dispersions of the nanocomposites, subsequently spread on the copper grid surfaces and inserted into the electron transmission microscope. 

The porosimeter (ASAP 2020 V4.01 H, Micromeritics Instrument Corporations, Norcross, GA, USA) available at the School of Chemical Sciences, Universiti Sains Malaysia, was employed to perform nitrogen adsorption–desorption analyses on the samples. The multilayer adsorption isotherm plots obtained by applying the Brunauer-Emmett-Teller (BET) equation to the resulting nitrogen physisorption data were used to determine the specific pore volume, pore size and surface area of the dried powdered nanocomposite samples. The samples were degassed at 383 K for 6 h under vacuum before sample analysis and data collection.

## 3. Results and Discussion

### 3.1. Spectroscopic and Surface Characteristics of the Fe_3_O_4_@C Nanocomposites

The surface properties of the three as-synthesised nanocomposite samples Fe_3_O_4_@C-T_190_t_3_, Fe_3_O_4_@C-T_190_t_4_, and Fe_3_O_4_@C-T_190_t_5_ were evaluated following a one-factor-at-a-time (OFAT) design approach using various complementary instrumental methods of analysis.

#### 3.1.1. Fourier Transform Infrared Spectroscopy

**Figure 1:**
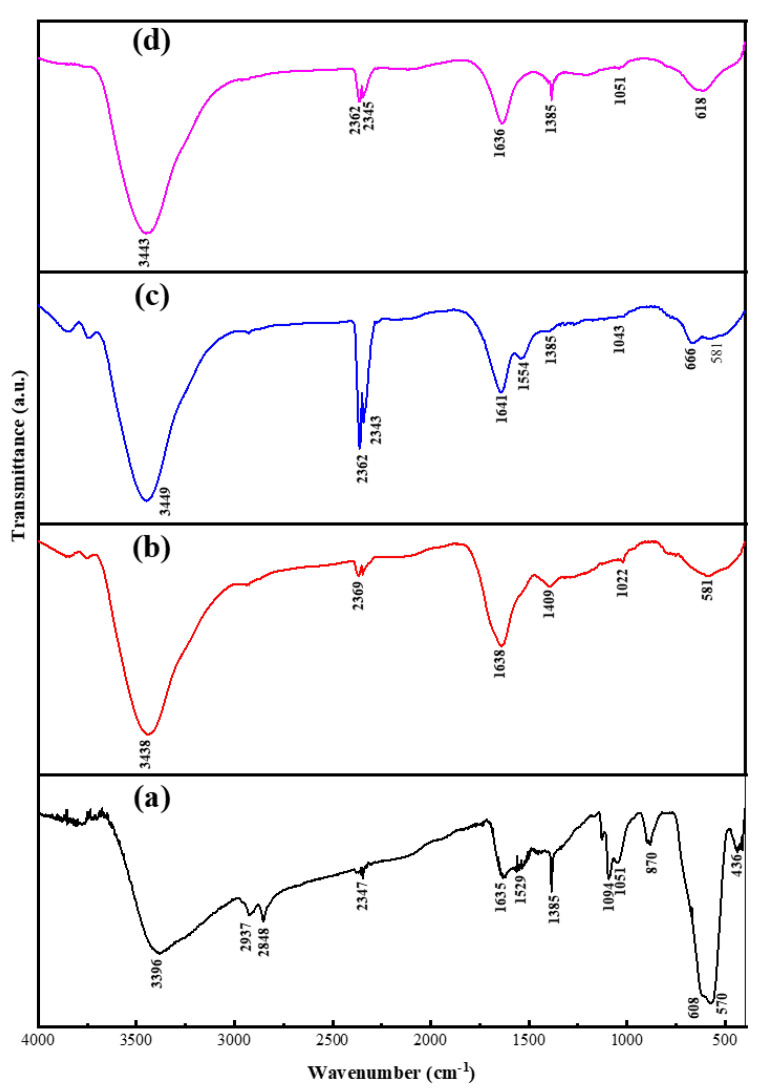
FTIR spectra for (**a**) Fe_3_O_4_ NPs; (**b**) Fe_3_O_4_@C-T_190_t_3_; (**c**) Fe_3_O_4_@C-T_190_t_4_; (**d**) Fe_3_O_4_@C-T_190_t_5_.

Figure 1 illustrates the FTIR spectra of the as-synthesised Fe_3_O_4_ NPs and Fe_3_O_4_@C nanocomposites. Assignments of the major absorption bands observed at 436 and 570 cm^−1^ in the spectrum of the parent magnetite NPs (a) are the characteristic peak of the intrinsic stretching vibrations modes for Fe–O bonds at the octahedral and tetrahedral cubic ferrite sites, respectively [30,31,32]. Meanwhile, the bands at 3396, 2347 and 1635 cm^−1^ are assigned to OH vibrations at the surface of the as-prepared magnetite NPs. The OH groups were inherited from the ethylene glycol molecules [33,34,35] and has higher vibrational intensities in Fe_3_O_4_ NPs over Fe_3_O_4_@C. The reduced vibrational intensities of these bands in Fe_3_O_4_@C indicate that, during glucose carbonisation, the carbon composited and effectively shielded the OH groups [36]. The peaks at 2848 and 2937 cm^−1^ are attributed to the antisymmetric and symmetric stretching vibration of C–H bonds introduced by ethylenediamine molecules at the solvothermal step for Fe_3_O_4_ NPs fabrication [37,38]. Weak to medium intensity peak at the 1385 cm^−1^ was ascribed to the antisymmetric C–N stretching vibrations coupled with the out-of-plane H–NH and N–H vibrational modes [33]. The peak at 870 cm^−1^ was assigned to the in-plane and out-of-plane vibrational modes for residual C–H bonds deformation [34]. Several peaks disappeared, shifted to new wavenumbers, or were retained in the resulting nanocomposites. Negligible shifts recorded for Fe–O bonds at the tetrahedral cubic ferrite sites and OH vibrations to 581 cm^−1^ and 3438–3449 cm^−1,^ respectively, indicate that the three nanocomposites retained the spectral characteristics of parent Fe_3_O_4_ NPs. The disappearance and shifting of some of the peaks in the spectra of the nanocomposites indicate the strong interaction between the parent Fe_3_O_4_ NPs core and the encapsulating carbon layer bounding its surface [35].

#### 3.1.2. X-ray Diffraction Analysis

**Figure 2:**
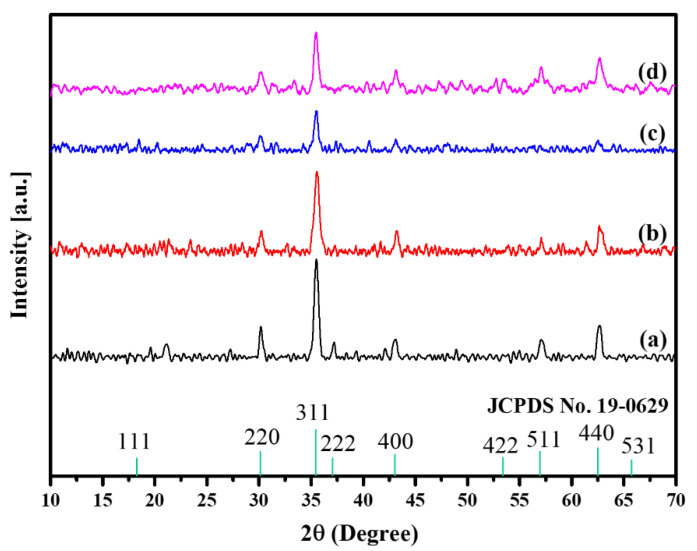
X-ray diffraction patterns of (**a**) Fe_3_O_4_ NPs; (**b**) Fe_3_O_4_@C-T_190_t_3_; (**c**) Fe_3_O_4_@C-T_190_t_4_; (**d**) Fe_3_O_4_@C-T_190_t_5_.

Crystal phases of the as-synthesised nanocomposites were investigated using an X-ray diffractometer, and the results are shown in Figure 2. The XRD patterns of Fe_3_O_4_@C-T_190_t_3_, Fe_3_O_4_@C-T_190_t_4_, and Fe_3_O_4_@C-T_190_t_5_ have diffraction peaks that matched the standard diffractogram of the magnetic Fe_3_O_4_ NPs (JCPDS No. 19-0629). The XRD peaks and crystal lattice constant (a_o_) in all the XRD patterns with the magnetite standards information indexed in the ICDD database [35] were compared. The a_o_ values determined from the peaks with the highest intensity for Fe_3_O_4_@C-T_190_t_3_, Fe_3_O_4_@C-T_190_t_4_, Fe_3_O_4_@C-T_190_t_5_ were found to be 8.38, 8.37 and 8.38 Å, in agreement with the standard parameter for the magnetite (8.39 Å). The diffraction peaks at 2θ = 57.1°, 62.7°, 53.5°, 43.1°, 35.5°, 30.2° and 21.9° which can be assigned to the Miller indices planes (511), (440), (422), (400), (311), (220) and (111), respectively, corresponding to a cubic inverse spinel unit cell structure of Fe_3_O_4_ nanomaterials [36,37] were retained in all three Fe_3_O_4_@C nanocomposites. The ability of the nanocomposites to retain the characteristic Miller indices planes identified in the parent Fe_3_O_4_ NPs after the synthesis is an indicator of their phase purity with respect to the Fe_3_O_4_ NPs. The XRD patterns of the Fe_3_O_4_@C nanocomposites did not show sharp peaks at 2θ values of 26° and 54°, which are the characteristic peaks for crystalline carbons [38,39]. However, the broad background, typical of amorphous material, in the XRD diffractograms of the nanocomposites relative to that of the Fe_3_O_4_ NPs confirmed the amorphous nature of the encapsulating carbon layer [39,40,41] is also absent. Thus, the XRD peaks corresponding to the encapsulating carbons were not found in the XRD pattern because they were poorly crystallised [42]. While the nature of the carbon layer may be ambiguous, the presence of the carbon layer in the nanocomposites is evidenced, especially from the thermal and EFTEM analysis.

The crystallite sizes (D*_hkl_*) of the nanocomposite samples were determined using the well-known Scherrer equation (Equation (1)):

(1) $$D_{hkl}=\frac{K\lambda }{\beta \cos\theta }$$

where  θ is the half diffraction angle of 2θ, K is the constant (=0.94), λ is the wavelength (=0.15406 nm), β is the full width at half maximum (FWHM) value of XRD diffraction peaks. The average crystallite size (D*_XRD_*) for each nanocomposite sample was calculated by dividing the total D*_hkl_* values by the number of individual planes [26]. The average crystallite grain size (D*_XRD_*) for each of the three nanocomposites (Fe_3_O_4_@C-T_190_t_3_, Fe_3_O_4_@C-T_190_t_4_, Fe_3_O_4_@C-T_190_t_5_) was computed to be 13 ± 8, 11 ± 6 and 16 ± 5 nm, respectively. The variation in the D*_XRD_* values in the nanocomposites can be attributed to the extent of carbonisation of the carbons composited to the parent Fe_3_O_4_ NPs at different reaction times, which was also in line with the reported EFTEM outcomes. Previously, Xu et al. [43] reported similar values for the average Fe_3_O_4_ NPs crystallite size of their as-synthesised Fe_3_O_4_@C nanocomposites.

#### 3.1.3. Thermogravimetric Analysis/Differential Thermogravimetry (TGA/DTG)

**Figure 3:**
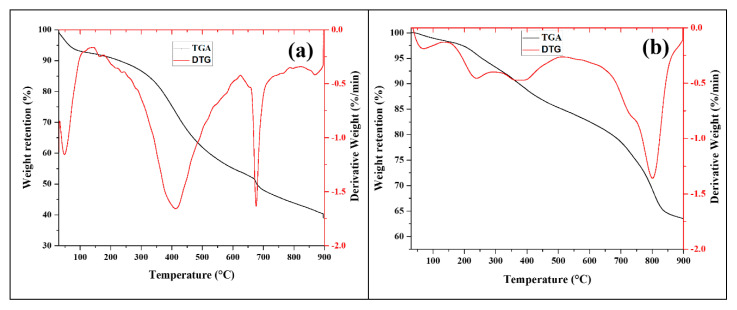

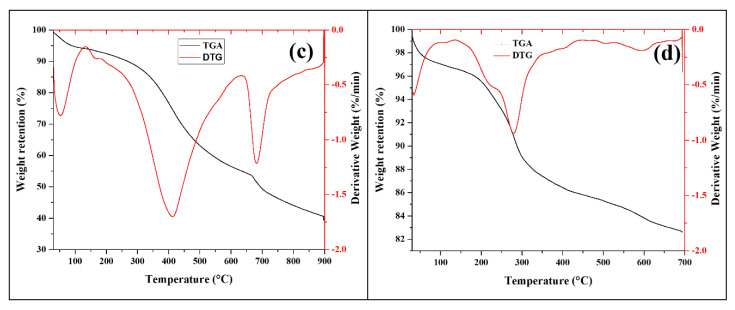
TGA/DTG curves for (**a**) Fe_3_O_4_@C-T_190_t_3_; (**b**) Fe_3_O_4_@C-T_190_t_4_; (**c**) Fe_3_O_4_@C-T_190_t_5_; (**d**) Fe_3_O_4_ NPs.

The thermograms of the as-synthesised Fe_3_O_4_@C nanocomposites and parent Fe_3_O_4_ NPs are illustrated in Figure 3. The recorded weight loss at 30–100 °C in Fe_3_O_4_@C-T_190_t_3_ (6.92%), Fe_3_O_4_@C-T_190_t_4_ (1.03%), and Fe_3_O_4_@C-T_190_t_5_ (5.37%) can be attributed to the evaporation of the water molecules physisorbed onto the surface of the samples. Degradation of the organic residue could probably be the factor responsible for the weight loss of 5.72–6.43% recorded within the temperature range of 100–300 °C for Fe_3_O_4_@C nanocomposites. The combustion of the encapsulating carbon was concurrently accomplished between 150 to 450 °C. The intensified degradation of organic residues and the carbonisation of the top layer of the Fe_3_O_4_@C nanocomposite surfaces could be asserted as the factor responsible for the weight loss recorded within the temperature range of 300–450 °C for Fe_3_O_4_@C-T_190_t_3_ (19.11%), Fe_3_O_4_@C-T_190_t_4_ (6.43%), and Fe_3_O_4_@C-T_190_t_5_ (19.55%). The uptake of carbon deposits from the in situ carbonisations of glucose precursor molecules onto the parent Fe_3_O_4_ NPs was more favourable during the hydrothermal synthesis of Fe_3_O_4_@C-T_190_t_3_ and Fe_3_O_4_@C-T_190_t_5_ than for Fe_3_O_4_@C-T_190_t_4_. As a result, the carbon content of the encapsulating layers in Fe_3_O_4_@C-T_190_t_3_ and Fe_3_O_4_@C-T_190_t_5_ was higher than in Fe_3_O_4_@C-T_190_t_4_. At 300~450 °C, gasification of the encapsulated carbon layer of the nanocomposites occurs. The gasification rate of the encapsulating carbon layer per minute in Fe_3_O_4_@C-T_190_t_3_ and Fe_3_O_4_@C-T_190_t_5_ was higher than in Fe_3_O_4_@C-T_190_t_4_ (as shown in red by plots in Figure 3) due to the higher carbon content in the composites. Further weight loss at higher temperatures, i.e., 450–900 °C, for Fe_3_O_4_@C-T_190_t_3_ (27.63%), Fe_3_O_4_@C-T_190_t_4_ (23.34%), and Fe_3_O_4_@C-T_190_t_5_ (28.54%), could be assigned to intensified carbonisation of the nanocomposite topmost layer and probable total exposure of the magnetite core of the nanocomposites [44]. Based on the overall weight loss throughout the entire heating profile of 30–900 °C, Fe_3_O_4_@C-T_190_t_4_ (36.5%) is the most stable sample in agreement with the carbon content shown in EDX analysis below. The thermal stability of both Fe_3_O_4_@C-T_190_t_5_ and Fe_3_O_4_@C-T_190_t_3_ are relatively similar, with a weight loss of as much as 60.0%. Moreover, the magnetite NPs from which the composites were fabricated recorded a far lower weight loss of 17.4% within the heating profile of 30–700 °C. The corresponding Fe_3_O_4_ NPs weight contents in the nanocomposites were estimated to be ~63.5% for Fe_3_O_4_@C-T_190_t_4_ and ~40.3% for both Fe_3_O_4_@C-T_190_t_3_ and Fe_3_O_4_@C-T_190_t_5_ within the heating profile. Fe_3_O_4_ NPs weight contents indicate the encapsulating carbon content composited in each as-synthesised nanocomposite material. However, relatively higher estimations of Fe_3_O_4_ NPs content (95.5%), thus signifying lower carbon content, were reported by Xu et al. [43].

The TGA/DTG analysis was used to monitor the weight loss and thermal stability of the sample as the temperature increased. As the increased weight loss with temperature could not allow estimations of the parent Fe_3_O_4_ NPs, the EDX analysis was employed in the semiquantitative estimation of the Fe_3_O_4_ NPs weights present in each nanocomposites sample. The major compositional component of the composites, Fe_3_O_4_ NPs, is completely transformed to hematite within the temperature range of 600–675 °C. Thus, comparisons between the weight loss in the nanocomposites and the parent Fe_3_O_4_ NPs precursor were performed by truncating the temperature axes of the nanocomposites to 700 °C, making the difference in the studied temperature range among the nanomaterials inconsequential. Change in weight of the composites within the range 675–900 °C could be attributed to the continued gasification of the encapsulating carbon layer and further transformations of the parent Fe_3_O_4_ NPs core [45].

#### 3.1.4. Scanning Electron Microscopy

**Figure 4:**
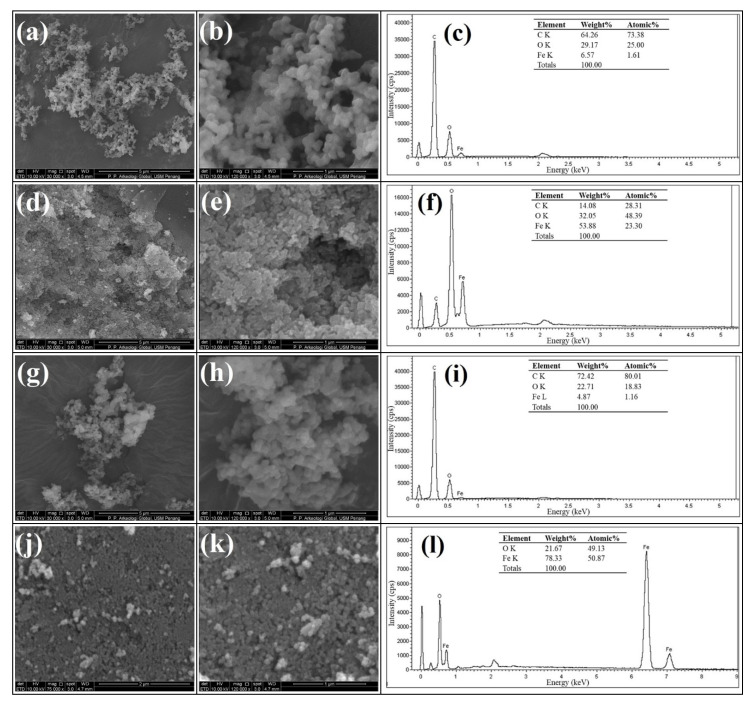
SEM images with different resolutions (30 k×, 120 k×) and EDX elemental composition of Fe_3_O_4_@C-T_190_t_3_ (**a**–**c**), Fe_3_O_4_@C-T_190_t_4_ (**d**–**f**), Fe_3_O_4_@C-T_190_t_5_ (**g**–**i**), and Fe_3_O_4_ NPs (**j**–**l**), respectively.

Figure 4a–l compares the SEM micrographs of the as-synthesised Fe_3_O_4_@C nanocomposites and Fe_3_O_4_ NPs at low and high magnifications together with their EDX spectra. The SEM micrographs of Fe_3_O_4_@C-T_190_t_3_ (Figure 4a,b) and Fe_3_O_4_@C-T_190_t_5_ nanocomposites (Figure 4g,h) revealed the conspicuous formation of well-defined clusters of nanospheres during the hydrothermal process. In contrast, Fe_3_O_4_@C-T_190_t_4_ nanocomposite (Figure 4d,e) exhibit the formation of agglomerated clusters among the nanospheres. The nanocomposites show varying degrees of distributions in particle size, uniformity, and aggregation [46]. The nanocomposites possess larger distribution of particle size (11–91 nm) than the parent Fe_3_O_4_ NPs (21–39 nm), as indicated by supporting information from EFTEM, which is comparable to the observation reported by Liu et al. [44].

The EDX spectroscopic analyses (Figure 4c,f,i) carried out on the nanocomposites indicated the proportion by weight of carbon to increase in the order Fe_3_O_4_@C-T_190_t_4_ (14.1%) < Fe_3_O_4_@C-T_190_t_3_ (64.3%) < Fe_3_O_4_@C-T_190_t_5_ (72.4%). It could be observed that Fe_3_O_4_@C-T_190_t_4_ has the lowest carbon content and recorded the least weight loss. Although it was found to compose of a typical stoichiometry near the standard phase composition of Fe_3_O_4_ NPs (78.3% Fe, 21.7% O) by atomic weight, the as-synthesised magnetite NPs obtained at the solvothermal step has been observed to exhibit an experimental stoichiometric ratio O:Fe (0.28) a little less than the expected value (0.38) [47]. This observed deviation can be attributed to the fact that EDX is a semiquantitative analytical technique. Previously, based on EDX analysis, Liu et al. [44] reported that their as-synthesised Fe_3_O_4_@C nanocomposites contained 15.4% carbon, which is in agreement with Fe_3_O_4_@C-T_190_t_4_ (14.1%) but far lower than those observed for Fe_3_O_4_@C-T_190_t_3_ (64.3%) and Fe_3_O_4_@C-T_190_t_5_ (72.4%). This observation indicates that the deposition of the encapsulating carbon layer onto the parent Fe_3_O_4_ NPs was more favourable at 5 h, and the least favourable reaction time was at 4 h.

#### 3.1.5. Transmission Electron Microscopy

**Figure 5:**
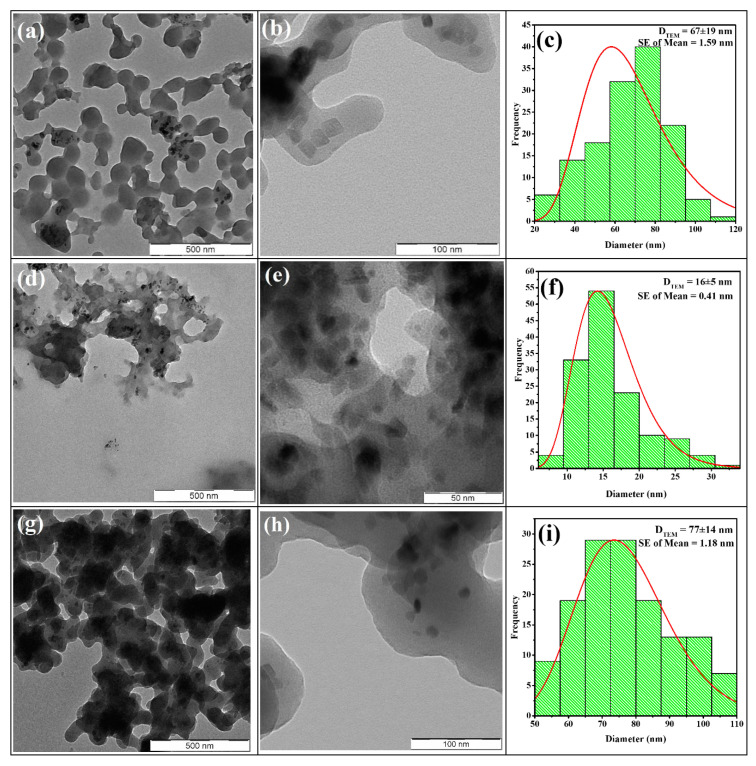

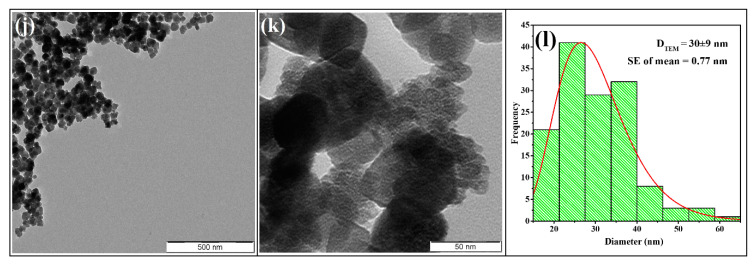
TEM images with different resolutions (12.5 k×, 100 k×) and particle size distributions of Fe_3_O_4_@C-T_190_t_3_ (**a**–**c**), Fe_3_O_4_@C-T_190_t_4_ (**d**–**f**), Fe_3_O_4_@C-T_190_t_5_ (**g**–**i**), and Fe_3_O_4_ NPs (**j**–**l**), respectively.

Evaluations of particle size distribution and further morphological elucidation of the nanocomposites were carried out using the EFTEM characterisation technique. Figure 5 demonstrates differing compositions of spherical, rod-like, and cubic morphologies depending on the reaction time (t_r_). The formation of nanocomposites with larger grain sizes has been conspicuously observed to be more favourable at t_r_ of 3 and 5 h, which in turn may be due to the amount of carbon in the nanocomposite. Figure 5a,d,g show the nanocomposites to appear as clusters of nanospheres while at higher magnifications (Figure 5b,e,h) they appear to be more cubic-like nanocomposites.

The nanocomposites’ particle size distributions were evaluated by fitting a lognormal distribution function over histograms constructed from the edge length (or diameter) data obtained via the measurements of 138 grains on the TEM micrograph of each sample. The Sturge’s rule, expressed in Equation (2), was applied to evaluate the number of bins (k) for the histograms [48,49]:

*k =* 1 + log_2_ *N* (2)

where N is the sample size representing the number of measurements carried out on each EFTEM micrograph. The results show that the mean particle diameter (D_TEM_) for the nanocomposites Fe_3_O_4_@C-T_190_t_3_, Fe_3_O_4_@C-T_190_t_4_ and Fe_3_O_4_@C-T_190_t_5_ were 67 ± 19, 16 ± 5, and 77 ± 14 nm, respectively, in contrast to the mean diameter of 30 ± 9 nm recorded for the parent Fe_3_O_4_ NPs (Figure 5l). Fe_3_O_4_@C-T_190_t_3_ and Fe_3_O_4_@C-T_190_t_5_ composites exhibited carbon shells of 37 and 47 nm in thickness, which were shells probably not observable in Fe_3_O_4_@C-T_190_t_4_ composite under the current microscopic settings as evidenced by its lowest carbon content from EDX analysis and lowest weight loss from the thermal analysis. Thus, the thickness of the carbon encapsulation at 3 h decreased at 4 h and then increased at 5 h, indicating that the thickness of the carbon layer from glucose resources can be controlled by increasing the reaction time [40]. The small standard error of the mean (0.41–1.59 nm) indicates that the values of the mean particle size (D_TEM_) are accurate and reliable representations of measurements in each sample size [50]. Furthermore, nanocomposites with similar particle size distribution (30 ± 19 nm) were also reported in the literature [44].

#### 3.1.6. Brunauer–Emmett–Teller (BET) Nitrogen Adsorption–Desorption Analysis

**Figure 6:**
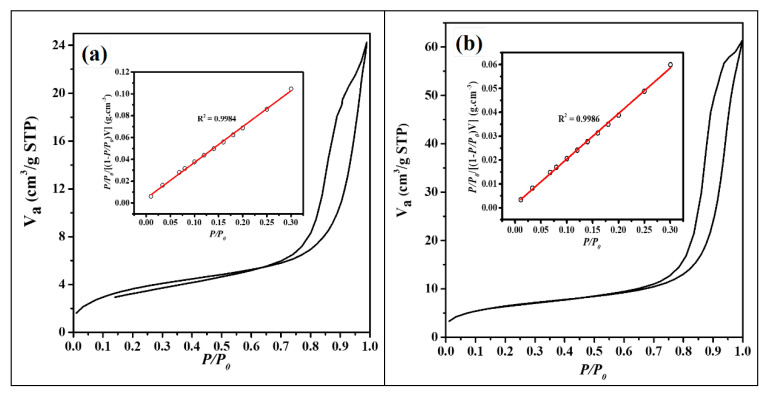

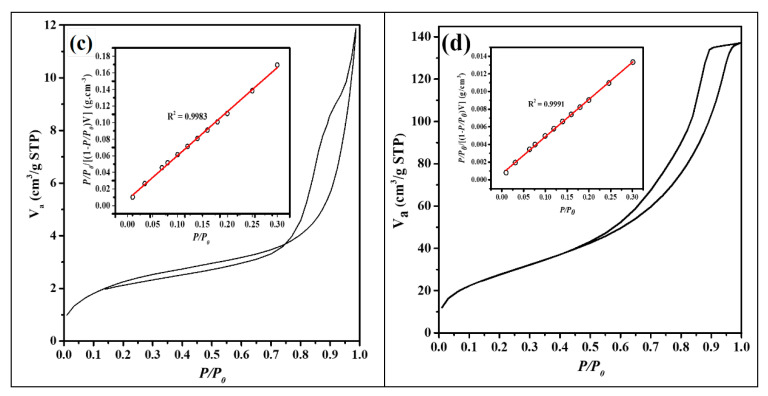
BET nitrogen adsorption–desorption hysteresis curves and linear isotherms (in the inset) of (**a**) Fe_3_O_4_@C-T_190_t_3_; (**b**) Fe_3_O_4_@C-T_190_t_4_; (**c**) Fe_3_O_4_@C-T_190_t_5_; (**d**) Fe_3_O_4_ NPs.

Figure 6 illustrates the comparison of the nitrogen adsorption–desorption isotherms for the as-synthesised Fe_3_O_4_@C nanocomposites and the magnetite NPs (Figure 6d). The BET isotherms of all the three nanocomposites exhibited an H3 hysteresis loop and were classified as Type II adsorption isotherms in line with International Union of Pure and Applied Chemistry (IUPAC). It is the characteristic of mesoporous materials whose mesopore volumes are not well defined because of the low-degree pore curvature and non-rigid structure of the aggregate nanocomposites [51,52,53]. The BET surface areas for the as-prepared magnetite composites are 12.39 m^2^ g^−1^ (Fe_3_O_4_@C-T_190_t_3_), 22.41 m^2^ g^−1^ (Fe_3_O_4_@C-T_190_t_4_), 7.61 m^2^ g^−1^ (Fe_3_O_4_@C-T_190_t_5_), compared to 5.6 m^2^ g^−1^ reported by Zeng and co-workers [20]. However, values as high as ∼342.7 m^2^ g^−1^ have been reported by Zhuang and co-workers [23].

**Figure 7:**
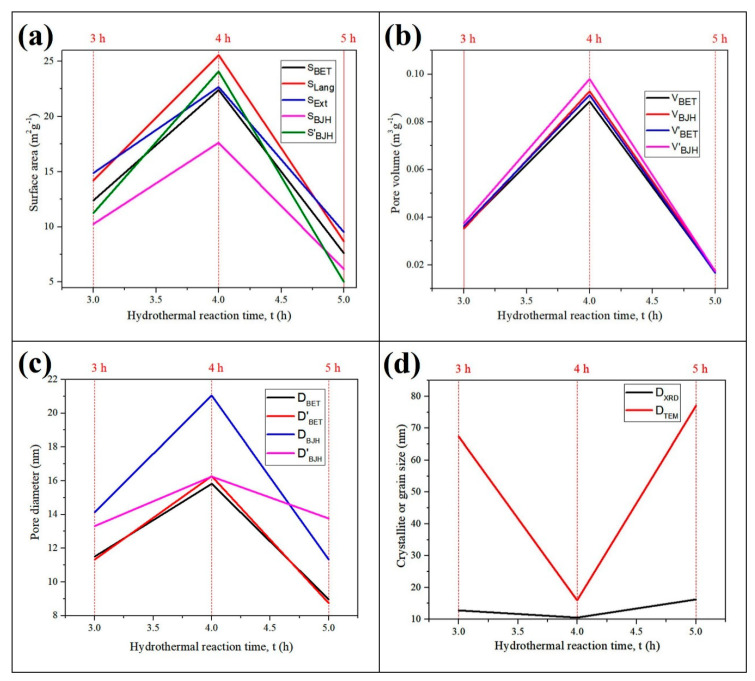
Effects of reaction time on the (**a**) surface area, (**b**) pore volume, (**c**) pore diameter and (**d**) crystallite or grain size of Fe_3_O_4_@C-T_190_t_3_, Fe_3_O_4_@C-T_190_t_4_ and Fe_3_O_4_@C-T_190_t_5_ nanocomposites obtained by hydrothermal synthesis.

Various surface properties of the nanocomposites were further compared (Figure 7a–d). Different forms of the surface area including the BET surface area (S_BET_), Langmuir surface area (S_Lang_), t-plot surface area (S_Ext_), Barrett-Joyner-Halenda (BJH) adsorption cumulative surface area of pores (S_BJH_) and BJH desorption cumulative surface area of pores (S’_BJH_) were compared in Figure 7a. Meanwhile, Figure 7b shows the comparison between the BET adsorption total pore volume (V_BET_) with BJH adsorption total pore volume (V_BJH_), BET desorption total pore volume (V’_BET_), and BJH desorption total pore volume (V’_BJH_) as pore volumes equivalents. In addition to that, the comparative distinction among BET adsorption average pore width (D_BET_), BET desorption average pore width (D’_BET_), BJH adsorption average pore width (D_BJH_), and BJH desorption average pore width (D’_BJH_) were presented in Figure 7c. Comparison between the mean crystallite size from XRD (D*_XRD_*) and mean particle size from TEM (D*_TEM_*) among the nanocomposites was presented in Figure 7d. The results show that Fe_3_O_4_@C-T_190_t_4_ has the highest surface area, total pore volumes and pore diameters and the smallest crystallite grain size and particle size among the three Fe_3_O_4_@C nanocomposites. However, the average surface area (114 m^2^ g^−1^), pore volume (0.21 cm^3^ g^−1^), and crystallite size (19 nm) of the parent Fe_3_O_4_ NPs were greater than those of the resulting composites. On the contrary, the average values of pore diameter and grain size of the parent Fe_3_O_4_ NPs (8.4, 30 nm) were both smaller than that of the Fe_3_O_4_@C-T_190_t_3_ (12.6, 67 nm) and Fe_3_O_4_@C-T_190_t_5_ (10.7, 77 nm) nanocomposites. Although the pore diameter of the Fe_3_O_4_ NPs (8.4 nm) was also less than that of the Fe_3_O_4_@C-T_190_t_4_ nanocomposite (17.4 nm), the grain size of the parent NPs (30 nm) was larger than in the nanocomposite (16 nm).

### 3.2. Two-Step Fe_3_O_4_@C NCs Formation Mechanism

The mechanism of the process was accomplished in two steps. The solvothermal method was used to synthesise the mesoporous Fe_3_O_4_ NPs in the first step, whose mechanism was proposed by Li et al. [29]. According to the mechanism proposed, EG is employed both as a reducing agent and high boiling point solvent. Sodium acetate is incorporated in the reaction mixture to act as a structure-directing agent, while ethylenediamine (EDA) is added as a chelating solvent to the parent Fe_3_O_4_ NPs [54]. Specifically, glycolaldehyde is produced by oxidising EG in solvothermal heating. The glycolaldehyde obtained reduces Fe^3+^ to Fe^2+^ ion, resulting in glyoxal. The acetate anions (CH_3_COO^−^) combine with the free Fe^3+^ and Fe^2+^ cations in the mixture to produce the acetates, Fe[CH_3_COO]_3_ and Fe[CH_3_COO]_2_, as the temperature rises. At such high temperatures, hydrolysis and alcoholysis convert Fe[CH_3_COO]_3_ and Fe[CH_3_COO]_2_ into Fe(OH)_3_ and Fe(OH)_2_. Dehydration of the hydrolytic products eventually produces the parent Fe_3_O_4_ NPs. As a chelating agent, EDA facilitates the growth of the encapsulating carbon particles on the Fe_3_O_4_ NPs under hydrothermal reaction to fabricate Fe_3_O_4_@C NCs samples at various reaction times [54].

### 3.3. Statistical Analysis of the Surface Properties

#### 3.3.1. Analysis of Variance (ANOVA)

Analysis of variance (ANOVA) was carried out to test the null hypothesis (H_0_) that all means for the surface area, total pore volume and pore diameter for the three nanocomposites were equal at the 0.05 statistical level of significance (α). The corresponding alternative hypothesis (H_1_) asserts that not all means of these surface properties of the nanocomposites were equal.

From the ANOVA results (Table 1), the hydrothermal t_r_ has been ascertained to affect the surface area significantly, total pore volumes and pore diameters for the three samples of the Fe_3_O_4_@C nanocomposites (*p* < 0.05). The summary of the model statistic (Table 2) was able to explain most of the variables to a great extent in total pore volumes (R^2^ = 99.60%) of the Fe_3_O_4_@C nanocomposites among the fabricated samples, followed by surface area (R^2^ = 90.13%). In comparison, the least variability was observed in the pore diameters (R^2^ = 69.83%).

#### 3.3.2. Pairwise Comparisons for Surface Properties

**Figure 8:**
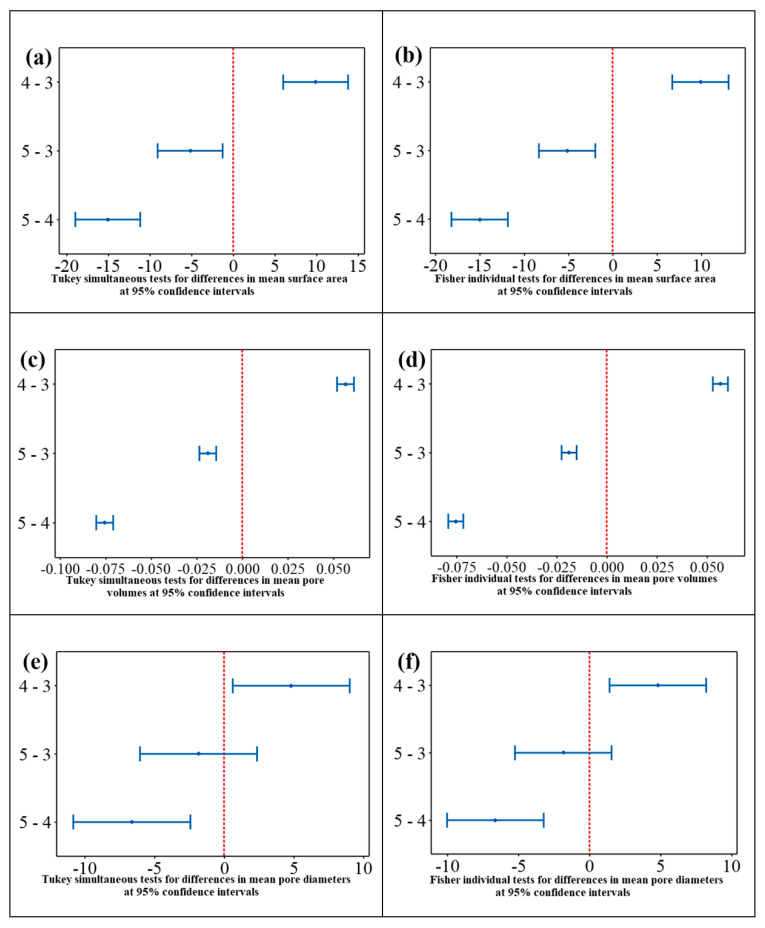
Tukey and Fisher tests of differences in means at 95% confidence intervals for (**a**,**b**) surface area, (**c**,**d**) pore volume, and (**e**,**f**) pore diameter of Fe_3_O_4_@C nanocomposite samples obtained at various hydrothermal reaction times, respectively.

Figure 8 presents the plots for the Tukey simultaneous tests and Fisher individual tests for pairwise differences between means surface properties (surface areas, pore volumes, pore diameters) of the nanocomposite samples obtained at various hydrothermal t_r_ values (Fe_3_O_4_@C-T_190_t_3_, Fe_3_O_4_@C-T_190_t_4_ and Fe_3_O_4_@C-T_190_t_5_) at 95% confidence intervals (CIs).

The confidence intervals (CIs) observed in the Tukey test plots (Figure 8a,c,e) for the difference in the pore diameter means ((0.59, 8.98), (−6.07, 2.33), (−10.85, −2.45)), total pore volume means ((0.05193, 0.06122), (−0.02365, −0.01435), −0.08022, −0.07093)), surface area means ((5.98, 13.77), (−9.08, −1.28), (−18.95, −11.16)) between the nanocomposites Fe_3_O_4_@C-T_190_t_4_ and Fe_3_O_4_@C-T_190_t_3_ (4–3), Fe_3_O_4_@C-T_190_t_5_ and Fe_3_O_4_@C-T_190_t_3_ (5–3), and Fe_3_O_4_@C-T_190_t_5_ and Fe_3_O_4_@C-T_190_t_4_ (5–4) do not include zero in their interval except for the difference in the pore diameter means (−6.07, 2.33) between Fe_3_O_4_@C-T_190_t_5_ and Fe_3_O_4_@C-T_190_t_3_ (5–3), respectively. Similarly, the observed confidence intervals (CIs) in the Fisher test plots (Figure 8b,d,f) for the difference in the means of pore diameter ((1.38, 8.19), (−5.27, 1.53), (−10.05, −3.25)), the means for the total pore volume ((0.05281, 0.06034), (−0.02276, −0.01524), (−0.07934, −0.07181)), the means of the surface area ((6.69, 13.06), (−8.36, −2.00), (−18.24, −11.87)) between the nanocomposites Fe_3_O_4_@C-T_190_t_4_ and Fe_3_O_4_@C-T_190_t_3_ (4–3), Fe_3_O_4_@C-T_190_t_5_ and Fe_3_O_4_@C-T_190_t_3_ (5–3), and Fe_3_O_4_@C-T_190_t_5_ and Fe_3_O_4_@C-T_190_t_4_ (5–4) exclude zero in their interval with exception to the difference in the pore diameter means (−5.27, 1.53) between Fe_3_O_4_@C-T_190_t_5_ and Fe_3_O_4_@C-T_190_t_3_ (5–3), respectively. These outcomes indicate that for all the pairwise comparisons under Tukey and Fisher tests, the mean surface properties between the nanocomposite samples are significantly different statistically except for the mean total pore volumes between nanocomposites Fe_3_O_4_@C-T_190_t_5_ and Fe_3_O_4_@C-T_190_t_3_ (5–3).

Table 3 tabulates the t values, individual confidence levels, adjusted *p* values, and simultaneous confidence levels from the Tukey and Fisher tests for all the pairwise differences in means for pore diameter, total pore volume and surface area of the nanocomposites. The t value test statistic measures the ratio between the difference in means of the surface properties and their standard errors in both the pairwise Tukey and Fisher tests between the nanocomposites. Observed absolute t values in both Tukey and Fisher tests for the difference in the pore diameter means (|3.18|, |1.24|, |4.42|), total pore volume means (|34.02|, |11.42|, |45.44|), surface area means (|6.76|, |3.54|, |10.30|) between the nanocomposites; Fe_3_O_4_@C-T_190_t_4_ and Fe_3_O_4_@C-T_190_t_3_ (4–3), Fe_3_O_4_@C-T_190_t_5_ and Fe_3_O_4_@C-T_190_t_3_ (5–3), and Fe_3_O_4_@C-T_190_t_5_ and Fe_3_O_4_@C-T_190_t_4_ (5–4) were found to be greater than their corresponding critical t values of 3.18, 3.18 and 2.78, respectively. This disparity indicates that the null hypothesis (H_0_) that the mean properties are the same should be rejected, and the alternative hypothesis (H_1_) that they are different should be accepted for all the nanocomposites in the pairwise comparative tests except for the difference in the pore diameter means between Fe_3_O_4_@C-T_190_t_5_ and Fe_3_O_4_@C-T_190_t_3_ (5–3). Since the absolute t values (|1.24|) for the difference in the pore diameter means between Fe_3_O_4_@C-T_190_t_5_ and Fe_3_O_4_@C-T_190_t_3_ (5–3) were less than the critical t values (3.18), the H_0_ should be accepted.

The simultaneous confidence levels (SCLs) for pore diameter (88.66%), pore volume (88.66%) and surface area (88.44%) indicate that we can be 88.66%, 88.66% and 88.44% confident that all the corresponding confidence intervals contain the true difference for specific pairwise comparison of the nanocomposite properties. Meanwhile, the individual confidence levels (ICLs) for pore diameter (97.91%), pore volume (97.91%) and surface area (97.94) indicate that we can be 97.91%, 97.91%, and 97.94% confident that each of the corresponding confidence intervals contains the true difference.

The adjusted *p* values (p-adj) observed in both the Tukey and Fisher tests for the differences in means of the surface properties show that all the properties between the compared pairs of nanocomposites were statistically different (p-adj < 0.05), except for the difference in the mean total pore volumes between nanocomposites Fe_3_O_4_@C-T_190_t_5_ and Fe_3_O_4_@C-T_190_t_3_ (5–3), which is found to be statistically inconsequential (p-adj > 0.05).

The reaction time-dependent hydrothermal synthesis performed for surface properties optimisation produced mesoporous as-synthesised nanocomposites with high volume-specific surface area (VSSA) values. The order of the VSSA values for the nanocomposites are Fe_3_O_4_@C-T_190_t_5_ (4.48 × 10^−8^) > Fe_3_O_4_@C-T_190_t_3_ (3.48 × 10^−8^) > Fe_3_O_4_@C-T_190_t_4_ (2.53 × 10^−8^). Large VSSA generally imparts nanomaterials unique surface characteristics, including enhanced thermal, electrical, mechanical, and optical properties [55]. Moreover, the Fe_3_O_4_@C nanocomposites could be biocompatible with high response electrochemical activity, thermal stability, and enhanced thermal and electrical conductivity [56]. Such carbon-encapsulated magnetite nanomaterials have the potential for diverse applications in various fields. They can be used as drug carriers in targeted drug delivery therapeutic systems [57,58], anodes materials in the construction of electrochemical lithium-ion batteries [59], microwave absorbing material for the treatment of microwave radiation pollution [60,61,62,63], and coolants for heat transfer applications in various systems (e.g., automobile radiators, refrigerators, electronic devices, solar energy heaters, etc.) [56]. Fe_3_O_4_@C can also be applied as photothermal contrasting-agents in proton magnetic resonance imaging for cancer treatment [64], peroxidase enzymatic mimics for glucose sensing in human body fluids [65], and in the remediation of wastewater containing recalcitrant organic compounds either as adsorbent material [66,67] or as the catalyst [68,69]. The utilisation of Fe_3_O_4_@C nanocomposites as the catalysts for treating recalcitrant pollutants in palm oil mill effluent (POME) by advanced oxidation processes (AOPs) is a potential area for application of the nanocomposites.

## 4. Conclusions 

Hydrothermal fabrications of carbon-encapsulated magnetite nanocomposites under a fixed temperature (190 °C) at reaction time (t_r_) of 3 h (Fe_3_O_4_@C-T_190_t_3_), 4 h (Fe_3_O_4_@C-T_190_t_4_), and 5 h (Fe_3_O_4_@C-T_190_t_5_) have been accomplished. The influences of t_r_ on mean surface properties (surface area, total pore volumes and pore diameters) of the nanocomposites were evaluated using one-way analyses of variance (ANOVA), Tukey simultaneous tests and Fisher individual tests for differences in mean properties of nanocomposite pairs. The ANOVA test shows that the hydrothermal reaction time (t_r_) significantly affected the surface area, total pore volumes and the particles size of the three Fe_3_O_4_@C nanocomposites samples (*p* < 0.05), and the model could explain almost 100%, 90% and 70% of the variability in the total pore volumes, surface area and pore diameters of the fabricated Fe_3_O_4_@C nanocomposites, respectively. These absolute t values from the pairwise comparison of the nanocomposites using Tukey and Fisher tests indicate the mean surface properties of nanocomposite pairs to be significantly different except for the mean total pore diameter of the pair Fe_3_O_4_@C-T_190_t_5_–Fe_3_O_4_@C-T_190_t_3_ (5-3). The adjusted *p* values (p-adj < 0.05) observed in the ANOVA for the pairwise difference in the mean properties further corroborated the outcome.

The levels of the mesoporosity, monodispersity, shape-controllability, and stability of the as-synthesised Fe_3_O_4_@C nanocomposite samples were established using FTIR, XRD, TGA/DTG, SEM/EDX, EFTEM and BET adsorption–desorption analyses. The as-synthesised nanocomposites largely retained the characteristic FTIR vibrations of the major absorption bands observed for Fe–O bond vibrations at the octahedral (436 cm^−1^) and tetrahedral (570 cm^−1^) cubic ferrite sites in the parent magnetite NPs with a shift in the latter band to 581 cm^−1^ and the shift in the OH vibrational assignments from 3396 to 3438–3449 cm^−1^. The Fe_3_O_4_@C-T_190_t_5_ nanocomposite (16.3 ± 5.3 nm) recorded the largest mean for crystallite size, followed by Fe_3_O_4_@C-T_190_t_3_ (12.8 ± 7.8 nm) and finally Fe_3_O_4_@C-T_190_t_4_ (10.5 ± 6.4 nm). The TGA/DTG analyses revealed the Fe_3_O_4_@C-T_190_t_4_ nanocomposite to have the highest thermal stability with the lowest weight loss (36.5%). In comparison, Fe_3_O_4_@C-T_190_t_5_ and Fe_3_O_4_@C-T_190_t_3_ were less stable to heat with the higher weight loss (59.8–60.1%) in agreement with the carbon content of the Fe_3_O_4_@C composites from EDX analysis.

## Figures and Tables

**Table 1:** Analysis of variance for the surface properties of the nanocomposites.

Source	DF ^1^	SS ^2^	MS ^3^	F Value	*p* Value
Surface area					
Reaction time, t_r_ (h)	2	585.25	292.626	54.79	0.000
Error	12	64.09	5.341		
Total	14	649.34			
Total pore volume					
Reaction time, t_r_ (h)	2	0.012364	0.006182	1117.55	0.000
Error	9	0.000050	0.000006		
Total	11	0.012414			
Pore diameter					
Reaction time, t_r_ (h)	2	94.19	47.093	10.42	0.005
Error	9	40.68	4.520		
Total	11	134.87			

^1^ DF, degree of freedom; ^2^ SS, sum of squares; ^3^ MS, mean square.

**Table 2:** Model statistic summary for the surface properties of the nanocomposites throughout the hydrothermal reaction time profile.

Statistic	Surface Property		
	Surface Area	Total Pore Volume	Pore Diameter
S	2.31107	0.002352	2.12613
R^2^	90.13%	99.60%	69.83%
R^2^-adj	88.48%	99.51%	63.13%
R^2^-pred	84.58%	99.29%	46.37%

**Table 3:** Significance statistic for Fisher individual tests and Tukey simultaneous tests on surface properties of the Fe_3_O_4_@C nanocomposite samples.

DOL ^1^	DOM ^2^	DSE ^3^	T Value	Fisher Test		Tukey Test	
				ICL (%) ^4^	p-adj ^5^	SCL (%) ^6^	p-adj ^5^
Pore diameter							
4–3	4.79	1.50	3.18	97.91	0.011	88.66	0.027
5–3	−1.87	1.50	−1.24		0.246		0.460
5–4	−6.65	1.50	−4.42		0.002		0.004
Pore volume							
4–3	0.05658	0.00166	34.02	97.91	0.000	88.66	0.000
5–3	−0.01900	0.00166	−11.42		0.000		0.000
5–4	−0.07558	0.00166	−45.44		0.000		0.000
Surface area							
4–3	9.88	1.46	6.76	97.94	0.000	88.44	0.000
5–3	−5.18	1.46	−3.54		0.004		0.010
5–4	−15.06	1.46	−10.30		0.000		0.000

^1^ DOL, difference of levels; ^2^ DOM, difference of means; ^3^ DSE, SE of difference; ^4^ ICL, individual confidence level; ^5^ p-adj, adjusted *p* value; ^6^ SCL, simultaneous confidence level.
